# Appearance of congenital hand anomalies

**DOI:** 10.1177/1457496920903987

**Published:** 2020-02-28

**Authors:** Noora N. Nietosvaara, Antti J. Sommarhem, Jani M. Puhakka, Ruth E. S. Tan, Johannes Schalamon, Aarno Y. Nietosvaara

**Affiliations:** Department of Pediatric Orthopedics and Hand Surgery, New Children’s Hospital, Helsinki University Hospital, Stenbäckinkatu 9, Helsinki, 00290, Finland; Department of Pediatric Orthopedics and Hand Surgery, New Children’s Hospital, Helsinki University Hospital, Helsinki, Finland; Department of Pediatric Orthopedics and Hand Surgery, New Children’s Hospital, Helsinki University Hospital, Helsinki, Finland; Department of Hand and Reconstructive Microsurgery, National University Hospital, Singapore; Department of Pediatric Surgery, Medical University of Graz, Graz, Austria; Department of Pediatric Orthopedics and Hand Surgery, New Children’s Hospital, Helsinki University Hospital, Helsinki, Finland

**Keywords:** Upper extremity deformities, congenital, esthetics, child, disability evaluation, hand

## Abstract

**Background and objective:**

Impact of appearance of congenital hand anomalies has not previously been reported. The purpose of this study was to describe the common perception about how different congenitally malformed hands look.

**Methods:**

We developed a questionnaire in a game format to evaluate the appearance of different hands. Altogether 1450 (954 females) 4- to 84-year-old residents (296 children) of two European and one Asian (n = 102) country were asked to rate the appearance of different looking hands on a five-point pictorial Likert-type scale. Standardized photographs of the dorsal aspect of 17 different congenitally malformed non-operated hands and a normal hand were presented to respondents. Significance of age, gender, nationality, and profession of the respondents was assessed.

**Results:**

The respondents’ ranking order of the hands was nearly consistent. The normal hand (mean = 4.43, standard deviation = 0.85, Md = 5) and clinodactyly (mean = 4.37, standard deviation = 0.86, Md = 5) were perceived to have the best appearance. Symbrachydactyly (mean = 1.42, standard deviation = 0.68, Md = 1) and radial club hand (mean = 1.40, standard deviation = 0.68, Md = 1) received the lowest scores. Adults rated the appearance of hands higher than children regarding 14 hands, females higher than men regarding 15 hands, and Europeans higher than Asians in 4 hands (p < 0.05, respectively). Europeans rated four-finger hand (mean = 3.21, standard deviation = 1.18, Md = 3) better looking than six-finger hand (mean = 2.92, standard deviation = 1.18, Md = 3, p < 0.005), whereas Asians gave higher scores to six-finger hand (mean = 2.66, standard deviation = 1.26, Md = 3) compared to four-finger hand (mean = 2.51, standard deviation = 1.14, Md = 2). Medical doctors and nurses gave higher scores compared to the other profession groups, school children, and high school students in five hands (p < 0.05).

**Conclusions:**

A normal hand is perceived distinctly better looking than most congenitally different hands. Different malformations’ appearance was ranked very coherently in the same order despite of participants’ age, gender, nationality, or profession. Asians seem to prefer an additional digit to a four-finger hand.

## Introduction

The reported incidence of congenital upper limb malformations in population-based studies from Australia and Sweden is around 20/10,000^1,2^. Polydactyly is the most common type of upper limb anomaly, followed by congenital trigger thumb/digit, camptodactyly, and syndactyly (2). Ulnar polydactyly is the most common polydactyly with an incidence of 3.5/10,000 live births (2). Upper limb deficiencies comprise approximately one-fourth of all congenital malformations with an incidence of 5/10,000 live births in Finland^3^.

Most studies on congenital limb anomalies are retrospective case series reporting mainly functional results of surgical treatment. There are few comprehensive studies focusing on the impact of upper limb malformations for the child’s development or psychological wellbeing^4–9^. Public reactions and perception of the appearance of congenital upper limb anomalies have not previously been reported.

Esthetics of a hand’s appearance is poorly defined. According to Jakubietz et al. (10), the most important factors determining hand beauty are the anatomically correct proportions of the digits and palm as well as normal subcutaneous tissue. The dorsal side of the hand is more visible to other people than the palmar side and thus probably more important esthetically^10^. Finger length is considered an important feature determining attractiveness of the hand as well as shape averageness and skin smoothness^11^.

During the past years about half of all children born in Scandinavia with an upper limb anomaly have been treated surgically^2,3^. Traditionally the main goal of surgery has been to improve function of the hand. We believe that more emphasis should be given to the appearance of the hand and wanted to study the common perception of the appearance of a normal hand and different congenital hand anomalies.

## Material and Methods

We created a questionnaire in a game format for assessing the appearance of congenitally different hands. People were presented with photographs of untreated congenital hand anomalies (n = 17) as well as a normal hand (n = 1), twice in a random order. The appearance of the hand was rated using a five-point pictorial (smiley) Likert-type scale transformed in a scale from 5 to 1 (Supplement Figure 1). Respondents were asked to choose one out of five smileys for each hand that best described the hands appearance in their opinion, the smileys were placed beneath each hand in the same order. The respondents’ age, gender, nationality, and profession were registered (Table 1). The respondents were also asked if themselves, their children, or someone they knew had a limb anomaly.

**Table 1.** Respondents’ gender, age, and profession according to country.

Respondents	Gender		Age (years)		Profession				
	Female	Male	<18	⩾18	Pupils	Students	Physicians	Nurses	Other
Finland (n = 1292)	867	425	231	1061	159	277	465	91	300
Austria (n = 56)	33	23	19	37	8	14	11	7	16
Singapore (n = 102)	54	48	18	84	16	10	4	3	69

Preoperative photographs of Caucasian children’s hands treated in Helsinki Children’s Hospital (currently known as New Children’s Hospital) were chosen for the game. The background of all photographs was set to blue and the size of the hand was digitally edited to be uniform, without changing its original proportions using Adobe Photoshop CC 20.0.1 release (Adobe Inc., San Jose, CA, United States). All photographs showed the dorsum of the hand with the wrist in neutral position and the fingers extended. Skin tone was edited to be similar. The following conditions were selected: ulnar polydactyly (floating type), radial polydactyly, clinodactyly, camptodactyly, simple complete syndactyly of the third web space, thumb hypoplasia (Blauth type 3A and 5), transverse reduction at wrist level, radial club hand, ulnar club hand, six-finger hand, four-finger hand, three-finger hand, cleft hand, symbrachydactyly, congenital constriction band syndrome, and macrodactyly.

**Fig. 1.**
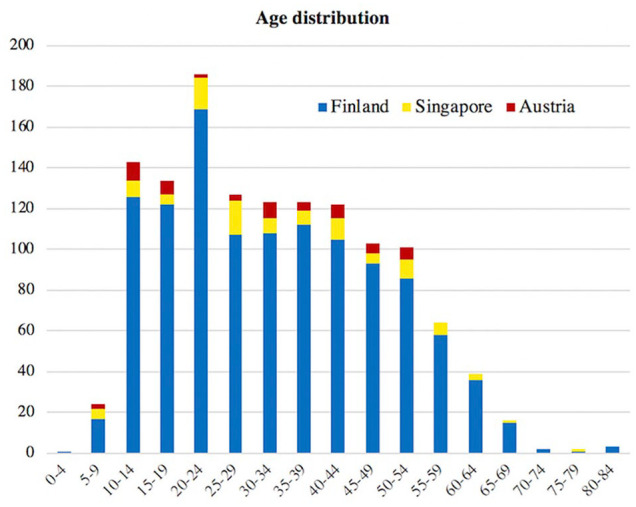
Age distribution of the respondents.

A total of 1450 (954 female) people participated in the study (1292 from Finland, 56 from Graz, Austria, and 102 from Singapore). In Finland, people were asked to participate in two randomly chosen schools and in public places as well as via email to two University hospitals. The first author conducted the survey in Austria and Singapore, where respondents were chosen randomly in Graz University Hospital and in the National University Hospital of Singapore. The respondents were between 4 and 84 years of age (Fig. 1) and comprised 183 school children and high school students, 301 university students, 480 medical doctors, 101 nurses, and 385 adults with miscellaneous professions, of which the biggest groups were architects (n = 33), office workers (n = 29), and teachers (n = 23). Of all respondents, 15 had a congenital limb malformation, 19 had a child, and 208 knew someone with a limb malformation.

In order to assess the repeatability of the evaluations by single raters, intraclass correlation (ICC) estimates and their 95% confidence intervals (CIs) were calculated for each hand based on single measure, absolute agreement, two-way mixed model. For interrater reliability, ICC estimates (with 95% CI) were calculated based on single measures, absolute agreement or consistency, two-way random model. Values less than 0.5 are considered to indicate poor reliability, 0.5–0.75 moderate reliability, 0.75–0.9 good reliability, and values greater than 0.90 excellent reliability^12^.

Mean scores of the two ratings for each hand were calculated after which a Kruskal–Wallis H test was used to calculate differences between the mean ranks of those mean scores. A post hoc test was used for pairwise comparison of the mean scores for the hands, as well as for analysis of nationality, profession, and association to limb anomalies of the respondents. Significance of age and gender was assessed using a Mann–Whitney U test. For the above-mentioned calculations, statistical analysis was performed using SPSS statistical package version 25 (SPSS Inc, Armonk, NY:IBM Corp, United States).

A Latent profile analysis was performed using version 3.5.0 in R package with a Mclust-function of the mclust package based on evaluations by the respondents in order to see if a grouping factor exists. A Gaussian finite mixture model fitted by expectation–maximization (EM) algorithm was used^13^. For all the statistical analysis, a p-value less than 0.05 was considered statistically significant.

## Results

**Fig. 2.**
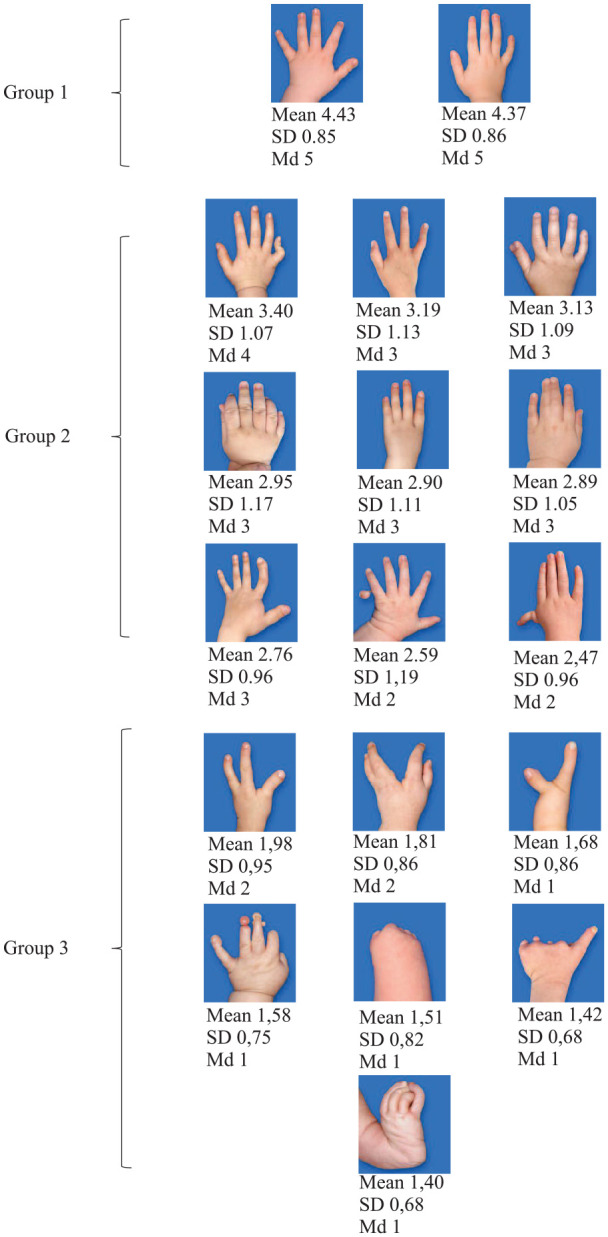
Hands divided into three subgroups using Latent profile analysis. Mean scores, standard deviations, and medians of all respondents below each photograph.

**Fig. 3.**
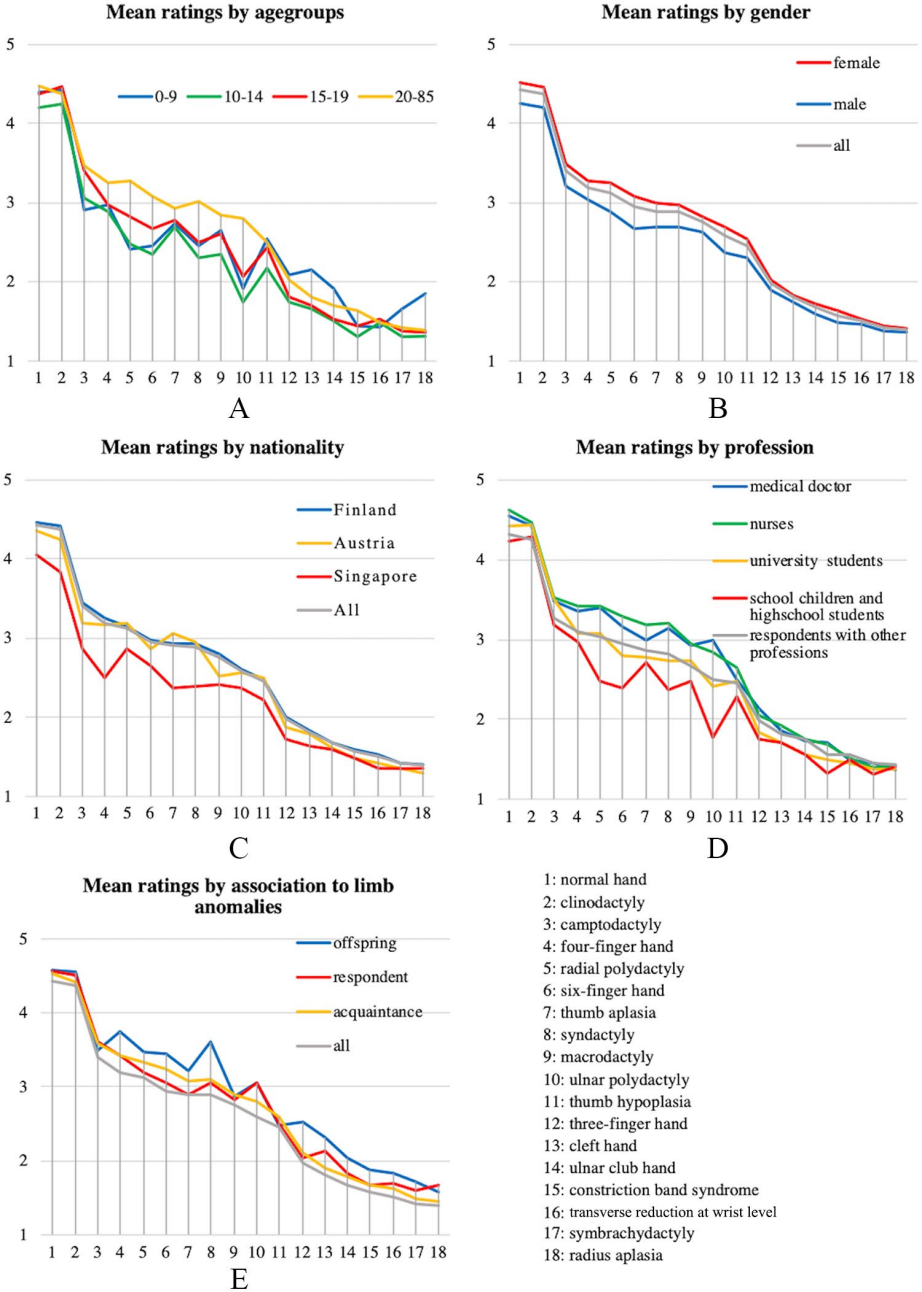
A) Difference between ratings of adults (n = 1154) and children and teenagers (n = 296). B) Difference between ratings of females (n = 954) and males (496). C) Difference between ratings of Finns (n = 1292), Austrians (n = 56), and Singaporeans (n = 102). D) Difference between ratings of respondents by profession: school children and high school students (n = 183); university students (n = 301); medical doctors (n = 480); nurses (n = 101); and respondents with other professions (n = 385). E) Difference between ratings of responders who know someone with a congenital limb malformation (n = 208), who’s child has a congenital malformation (n = 19), and responders with a congenital limb malformation (n = 15).

Hands with four separate fingers and a thumb of normal size (normal hand, clinodactyly, and camptodactyly) had the highest mean scores, whereas hands with most missing parts (transverse reduction at wrist level, monodactylous symbrachydactyly, radial club hand) had the lowest mean scores (Table 2). Perceived appearance of all hands was divided into three groups (mean score: >4, 2–4, and <2) using Latent profile analysis (Fig. 2). The intermediate group consisted of camptodactyly and hands with one missing or additional finger, or malformation of one or two rays. Appearance was perceived poor in hands with ⩽3 fingers, several abnormally developed parts, cleft, or obvious deformity. In general, all scores (n = 1450) were very consistent ranking most of the presented hands in the same order despite age, gender, nationality, or profession of the respondents (Fig. 3). Intrarater reliability was good (ICC = 0.86, 95% CI = 0.86–0.87, p < 0.001) and interrater reliability for absolute agreement and consistency was moderate (ICC = 0.52, 95% CI = 0.38–0.71, p < 0.001 and ICC = 0.67, 95% CI = 0.55–0.82, p < 0.001) according to Koo and Li (12). Evaluation was most coherent between respondents concerning the two best and the six poorest looking hands, these hands having the lowest standard deviation (<0.9). In subgroup, analysis of different respondent groups minor differences were, however, demonstrated (Fig. 3).

**Table 2.** Mean ratings by all respondents.

Picture	Mean	SD	Md
Normal hand	4.43	0.85	5
Clinodactyly	4.37	0.86	5
Camptodactyly	3.40	1.07	4
Four-finger hand	3.19	1.13	3
Radial polydactyly	3.13	1.09	3
Six-finger hand	2.95	1.17	3
Thumb aplasia	2.90	1.11	3
Simple complete syndactyly of the third web space	2.89	1.05	3
Macrodactyly	2.76	0.96	3
Ulnar polydactyly	2.59	1.19	2
Thumb hypoplasia	2.47	0.96	2
Three-finger hand	1.98	0.95	2
Cleft hand	1.81	0.86	2
Ulnar club hand	1.68	0.86	1
Constriction band syndrome	1.58	0.75	1
Transverse reduction at wrist level	1.51	0.82	1
Symbrachydactyly	1.42	0.68	1
Radius aplasia	1.40	0.68	1

SD: standard deviation.

Adults gave a higher mean score to almost all hands compared to adolescents (10–19 year olds), with the exception of transverse reduction at wrist level and clinodactyly (15–19 year olds only). The difference between the ratings of adults and adolescents was statistically significant concerning 13/18 (10–14 year olds) and 7/18 (15–19 year olds) of the presented hands. The discrepancy between adults’ and children’s (<9 years of age) mean scores was less obvious with adults giving higher scores to 11/18 (4/18, p < 0.05), the most significant difference between the mean scores was concerning radius aplasia where children rated it higher (1.86) than adults (1.39), p < 0.05. There was no difference found in the ratings of clinodactyly, transverse reduction at wrist level, and ulnar club hand, p > 0.05. Intrarater reliability was good for adults (ICC = 0.88, 95% CI = 0.87–0.88) and for 10- to 19-year-old adolescents and teenagers (ICC = 0.83, 95% CI = 0.82–0.84), but only moderate for <10-year-old children (ICC = 0.65, 95% CI = 0.6–0.7) (Fig. 3A).

Females (n = 954) rated all hands higher than males (n = 496). This difference was statistically significant in 15/18 hands and most profound concerning six-finger hand, ulnar polydactyly, and thumb hypoplasia (Fig. 3B). The ranking order of the hands differed only between syndactyly and six-finger hand. Females perceived six-finger hand better than syndactyly, whereas males ranked them the other way around.

There were no differences between the mean scores of Finnish and Austrian respondents, with the exception of macrodactyly (2.80 vs 2.53, p < 0.005), but Finns rated 12/18 hands significantly higher than Singaporeans (Fig. 3C). The difference was most profound concerning the four-finger hand (3.25 vs 2.51), camptodactyly (3.45 vs 2.87), and thumb aplasia (2.93 vs 2.37).

Regarding profession of the respondents, we compared ratings by school children and high school students, university students, medical doctors, and nurses to adults with other professions. School children and high school students gave the lowest ratings to nearly all hands (p < 0.05 in 5/18 hands). On the contrary, medical doctors and nurses, whose responses did not differ significantly, gave overall the highest ratings (p < 0.05 in 5/18 hands). Very small differences were found between the ratings of university students and adults with miscellaneous professions. Cleft hand, transverse reduction at wrist level, symbrachydactyly, and radius aplasia were rated very similarly by all (p > 0.05) (Fig. 3D).

Respondents who knew someone with a limb anomaly (n = 208) gave higher scores to all hands (p < 0.05 in 14/18 hands) compared to the overall ratings. Respondents with a limb anomaly (n = 15) or with a child with a limb anomaly (n = 19) gave even higher ratings for almost all hands (Fig. 3E), but these subgroups were too small for reliable statistical analysis.

## Discussion

To evaluate the social impact of a congenital hand anomaly, we have, for the first time, assessed how congenitally different non-operated hands are perceived by common people. We found that all congenitally different hands, except clinodactyly, are seen significantly less pleasing than the normal hand. Second, different malformations appearance was ranked almost coherently in the same order despite participants’ age, gender, nationality, or profession.

The aesthetic qualities of the hand are subjective, but it has been suggested that a beautiful hand appears youthful, healthy and has long, but proportionally appropriate fingers^10,11^. The factors that correlated negatively with the appearance of the hands in this study were abnormal number or length of fingers, the number of abnormally looking fingers, and obvious abnormality of the hand or wrist. Second, congenitally different hands with a somewhat intact symmetry were regarded as better looking than hands where the basic symmetry was disrupted. Hands with the lowest mean scores in our study, radial club hand, monodactylous symbrachydactyly, and transverse reduction at wrist level, had a combination of all these factors. A missing or obviously hypoplastic malaligned thumb was regarded as less aesthetic than a missing finger. Malformed or accessory parts were also consider esthetically so displeasing that thumb aplasia received a higher mean score than the hand with a type 3A hypoplastic thumb.

It has been shown that children are more critical regarding aesthetics compared to adults in a study on the desirable tooth color ^14^. This is in accordance with the present study since children aged 10–14 gave the lowest scores compared to older respondents. The biggest difference comparing ratings by adults compared to children (<19 years of age) was regarding ulnar polydactyly, children rating it significantly lower than adults. One explanation might be that adults could have had difficulties excluding a vision of a normal looking hand after excision of the floating extra digit. Children between 0 and 19 years of age gave clinodactyly a slightly higher mean score than the normal hand, which could be due to the fact that there was dirt under the fingernails of the child with the normal hand. The reason for children giving lower scores than adults is unknown, but it could indicate that children are more critical than adults concerning appearance.

There are conflicting data about gender differences in studies assessing satisfaction of cleft lip patients with postoperative facial appearance ^15^, but we could not find any previous research assessing possible gender differences on how congenitally malformed hands are seen. We cannot explain the reason for females giving higher mean scores to all hands than males. It is also unclear why a six-finger hand was seen better looking than syndactyly by females on contrary to male respondents.

It has been said that “Beauty is in the eye of the beholder” and is at least somewhat affected by religion and culture. We could not find any earlier research on how different cultural backgrounds might influence perception of the appearance of congenitally different hands. Most of the respondents in this study were Finnish representing a Nordic culture with protestant Christian roots compared to Central European Austrians with a strong influence of the Catholic Church. In contrast to these two European countries, Singaporeans represent a multicultural Asian society with a broad diversity of religions (Buddhism, Christianity, Islam, and Taoism). In general, Singaporeans scored the appearance of the presented hands consistently lower than Europeans. The biggest difference between Singaporean and Europeans responders was in the mean scores of four-finger hand and thumb aplasia. The reason for this discrepancy is unclear, but one possible explanation could be that it is fundamental in many Asians cultures that a body should be “whole,” without any missing parts.

Patients and their parents have been reported to be more satisfied with patients’ appearance following cleft surgery than laypeople^16^. This is in accordance to the results of this study that participants that knew someone with a limb anomaly saw congenitally different hands more beautiful than the rest. Participants with a limb anomaly or with an offspring with a limb anomaly appeared to give the highest scores for all hands in this questionnaire.

The appearance of photographs of congenital hand differences was assessed surprisingly uniformly in our questionnaire using a five-point pictorial (smiley) Likert-type scale. The instrument used is a study limitation, and a possible scoring bias in feasibility could not entirely be ruled out. The use of emoticons and emoji have become popular as stimulus materials in scientific research^17^, and the five-point pictorial (smiley) Likert-type scale has been validated for assessing pain in children^18^. Intrarater reliability was good concerning respondents ⩾10 years of age. Perceived appearance varied the least in hands with the best and the worst scores. The smallest differences between all respondents were found in the ratings of the hands that were perceived to have the poorest appearance. The results of this study should however be interpreted with some caution mainly because the evaluation was based on one dorsal photograph of each hand, although the eye catches a moving object better than a static one. A video showing each hand’s function might therefore have allowed a better assessment, because function of the hand does probably affect its appearance as suggested by Bellew and Kay^4^. Second, only one photograph of the most common hand differences were shown although anomalies are more or less different even within each subtype. Therefore, the ranking order, especially of the anomalies that were perceived moderately looking, cannot be generalized. Third, some of the presented photographs were taken of infants hands (e.g. radial club hand and constriction band syndrome hand) with more subcutaneous fat and more prominent skin creases than in the rest, which could affect the rating. Fourth, an adult’s finger is partially visible holding the six-finger hand in position and there was dirt under the fingernails of the child with a normal hand. Finally, the geographic distribution of the respondents is limited to Finland, Austria, and Singapore, with a relatively small number of respondents in the latter two.

Despite age, gender, or nationality, the appearance of different congenital hand malformations is perceived very similarly. Opinions vary the least concerning the best looking and the poorest looking hands. Singaporeans seem to prefer an extra digit to a hand with less than five fingers, whereas Europeans see the four-finger hand better looking than the six-finger hand.

## Supplemental Material

sj-png-1-sjs-10.1177_1457496920903987 – Supplemental material for Appearance of congenital hand anomalies Click here for additional data file. Supplemental material, sj-png-1-sjs-10.1177_1457496920903987 for Appearance of congenital hand anomalies by Noora N. Nietosvaara, Antti J. Sommarhem, Jani M. Puhakka, Ruth E. S. Tan, Johannes Schalamon and Aarno Y. Nietosvaara in Scandinavian Journal of Surgery
