# Supplementary figures and images for: Appearance of congenital hand anomalies
Source: Scand J Surg. 2020 Feb 28;110(3):434–40. doi: 10.1177/1457496920903987 (PMC8551435; doi:10.1177/1457496920903987)

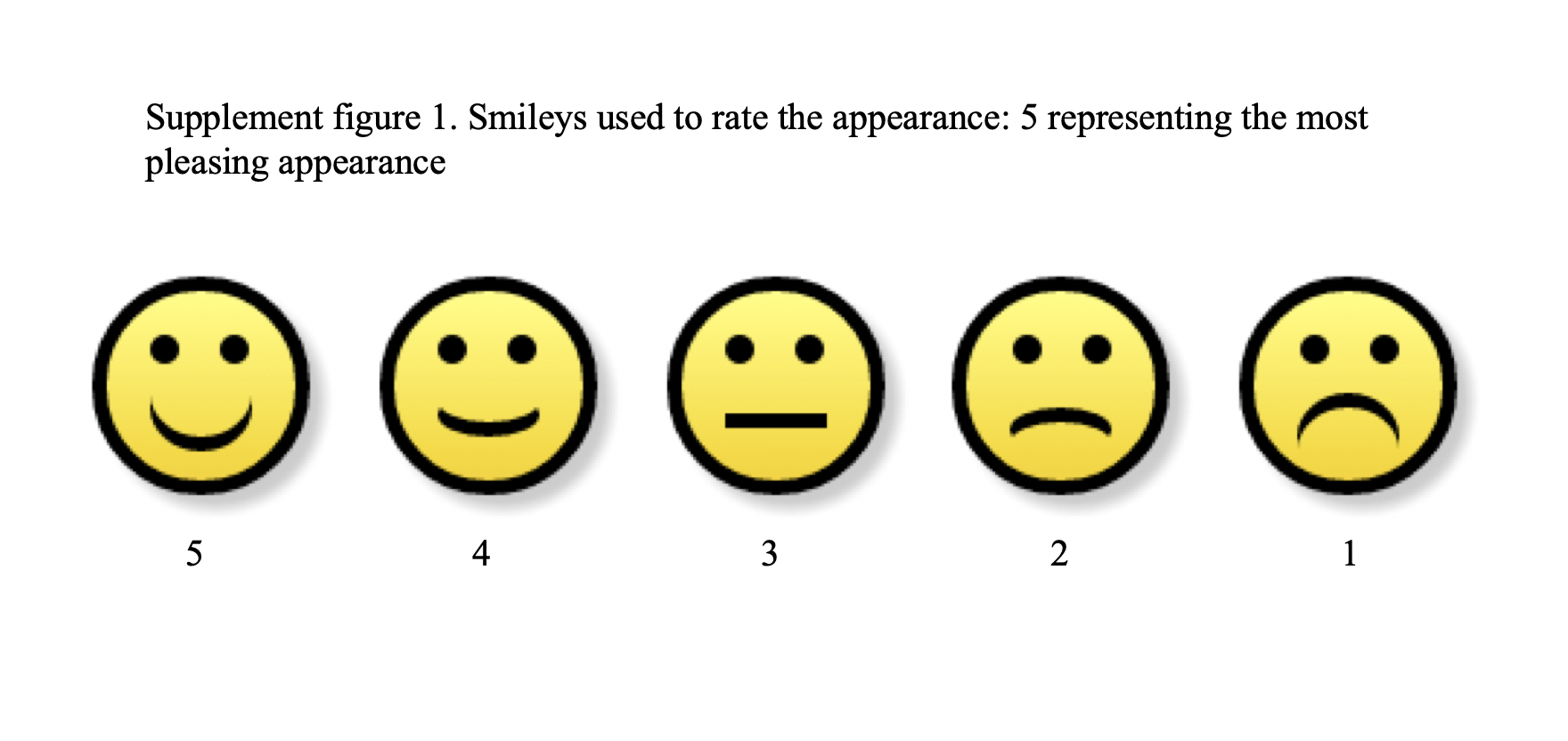

Supplement: sj-png-1-sjs-10.1177_1457496920903987 – Supplemental material for Appearance of congenital hand anomalies [file sj-png-1-sjs-10.1177_1457496920903987.png]
